# The clinical effectiveness of self-care interventions with an exercise component to manage knee conditions: A systematic review

**DOI:** 10.1016/j.knee.2015.05.003

**Published:** 2015-10

**Authors:** Kate Button, Paulien E. Roos, Irena Spasić, Paul Adamson, Robert W.M. van Deursen

**Affiliations:** a School of Healthcare Sciences, Cardiff University, United Kingdom; b School of Computer Science and Informatics, Cardiff University, United Kingdom; c Physiotherapy Department, Cardiff and Vale UHB, United Kingdom

**Keywords:** Self-care, Exercise, Knee, Rehabilitation, Patient education

## Abstract

**Objective:**

Treatment of knee conditions should include approaches to support self-care and exercise based interventions. The most effective way to combine self-care and exercise has however not been determined sufficiently. Therefore the aim was to evaluate the clinical effectiveness of self-care programmes with an exercise component for individuals with any type of knee conditions.

**Methods:**

A keyword search of Medline, CINAHL, Amed, PsycInfo, Web of Science, and Cochrane databases was conducted up until January 2015. Two reviewers independently assessed manuscript eligibility against inclusion/exclusion criteria. Study quality was assessed using the Downs and Black quality assessment tool and the Cochrane Risk of Bias Tool. Data were extracted about self-care and exercise intervention type, control intervention, participants, length of follow-up, outcome measures, and main findings.

**Results:**

From the 7392 studies identified through the keyword search the title and abstract of 5498 were screened. The full text manuscripts of 106 studies were retrieved to evaluate their eligibility. Twenty-one manuscripts met the inclusion/exclusion criteria.

**Conclusion:**

The treatment potential of combined self-care and exercise interventions has not been maximised because of limitations in study design and failure to adequately define intervention content. Potentially the most beneficial self-care treatment components are training self-management skills, information delivery, and goal setting. Exercise treatment components could be strengthened by better attention to dose and progression. Modern technology to streamline delivery and support self-care should be considered. More emphasis is required on using self-care and exercise programmes for chronic condition prevention in addition to chronic condition management.

## Highlights

• Limited supportive evidence for combined self-care and exercise interventions
• Training self-care skills, information delivery and goal setting are beneficial.
• Exercise prescription can be improved by attention to dose and progression.
• Self-care and exercise treatment needs development for chronic condition prevention.
• Modern technology could improve delivery and monitoring to inform best practice.

## 1 Introduction

Self-care is a concept widely applied across healthcare and can be broadly defined as “what people do for themselves to establish and maintain physical and emotional health and prevent or deal with minor illness, injury, or chronic conditions”. This incorporates concepts such as exercise, hygiene, nutrition, medication, and environmental and socioeconomic factors [1], [2]. Treatment techniques that have been incorporated into self-care programmes include: collaborative care plans between service users and healthcare professionals; setting goals that are reviewed and modified; helping individuals explore barriers to self-care; aiding people to monitor their symptoms and what action to take; providing advice and education; and coaching and peer support from other service users [3], [4]. For musculoskeletal conditions, self-care programmes have been developed and evaluated for knee osteoarthritis, but their effectiveness is considered limited, due to methodological weaknesses in study designs [5], [6].

Despite this, current evidence suggests that individuals with knee conditions should be given access to information about their condition and advice on self-management, especially exercise [6], [7]. This poses certain challenges to healthcare professionals that deliver exercise based interventions. Firstly, there is evidence within physiotherapy that information provision and exercise are the most widely used treatment modalities for knee rehabilitation [8], but much of the self-care and exercise research has been carried out independent of each other. Therefore, the most successful approaches of combining self-care and exercise are not yet known; it is also not clear whether the same self-care techniques benefit all knee conditions. Secondly, exercise rehabilitation is an effective intervention across all knee conditions, ranging from ruptures of the anterior cruciate ligament to patellofemoral joint pain and post-operative care following knee surgery [7]. Techniques on exercise prescription and progression are however less well developed for individuals with a knee condition. Finally, when modern technology, such as the Internet, is used to deliver supported self-care programmes, it needs to be clarified how effective it is for individuals with knee conditions.

Several systematic reviews and meta-analyses have been carried out to evaluate the effectiveness of self-care interventions (with or without exercise components), but these have been confined to patients with osteoarthritis [5], [9], [10], [11]. Many of these have also included other pathologies or were not knee specific [5]. One study evaluated self-management or exercise programmes but not necessarily studies that combined the two [11]. Overall these studies have concluded that the evidence was low to moderate quality and only demonstrated small benefit to patients. The current systematic review extends these themes to identify specific self-care and exercise approaches and outcome measurements that need to be developed to improve clinical effectiveness in the future. Therefore the aim was to evaluate the clinical effectiveness of self-care programmes with an exercise component for individuals with any type of knee condition. Clinical effectiveness was measured according to patient rated outcome measure scores.

## 2 Methods

The systematic review was carried out according to the PRISMA statement [12].

### 2.1 Data sources and search

The search strategy was designed by combining keywords from relevant published literature on self-care programmes and knee conditions [4], [7]. The first category of keywords related to knee conditions: knee injury, knee surgery, knee joint, knee osteoarthritis, osteoarthritis, knee dislocation, knee replacement, knee arthroplasty, anterior cruciate ligament, medial collateral ligament patellofemoral pain, and knee pain. The second category of keywords related to self-care: self-care, self-efficacy, internet, social support, social networking, patient education, telemedicine, behaviour therapy, goal setting, self-groups, self-monitoring, self-management education, and motivational interviewing. This keyword search was carried out on the following electronic databases: Medline, CINAHL, Amed, Embase, PsycInfo, Web of Science, and Cochrane Library Trials Register to search for manuscripts published up until January 2015 (no restriction was placed on the start date of the search). These searches were combined and limited to studies on adult humans and written in the English language. Additional articles were identified by reviewing the reference list of the retrieved manuscripts and checking the citations of all the full text manuscripts. An example of the search strategy is illustrated in Appendix A.

### 2.2 Study selection

Initially, all of the titles and abstracts were screened by reviewer 1 to check if they met the inclusion and exclusion criteria. To be included a study had to:

• Have an adult population aged 18 + years
• Be conducted in the out-patient/community setting
• Use individuals with knee conditions
• Be a randomised control trial
• Evaluate clinical effectiveness using patient rated outcome measures as a primary or secondary outcome measure
• Be an intervention that includes an exercise and self-care component
• Be written in the English language.

Studies were excluded if they:

• Evaluated economic effectiveness as the primary outcome
• Evaluated in-patient care or housebound individuals
• Compared lay versus professional intervention delivery
• Targeted other family members/partners
• Targeted treatment of other joints in the intervention or treatment to other health conditions in addition to the knee.

Full text manuscripts that met the inclusion/exclusion criteria were retrieved. For studies with a knee osteoarthritis population this could be unilateral or bilateral. Two reviewers checked the full text manuscripts against the inclusion/exclusion criteria, any disagreements were resolved through discussion to achieve consensus. The reference lists and citations of all full test articles were checked for relevant articles.

### 2.3 Quality assessment

The methodological quality of the remaining articles was independently rated by two reviewers using the Downs and Black quality assessment tool [13]. Any disagreements in the quality rating were resolved by discussion and reaching a consensus. The ‘Risk of Bias’ tool evaluated in depth any sources of manuscript bias [14].

### 2.4 Data extraction

Data were extracted about research design, self-care intervention, control intervention, participants, follow-up, main findings, and outcome measures. All data were extracted by reviewer 1 and then checked by reviewer 2, agreement was achieved by consensus.

## 3 Results

### 3.1 Study characteristics

**Fig. 1:**
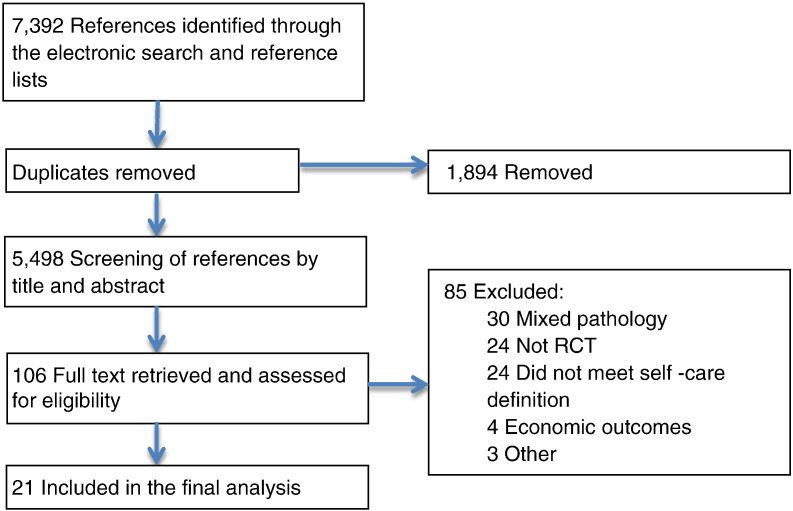
Flow chart showing the selection process of studies included in the systematic review.

From the n = 7392 articles identified through the literature search n = 21 were included in the systematic review. Of these n = 18 were related to self-care interventions and three were long-term follow-ups of these interventions [15], [16], [17]. The PRISMA flow chart (Fig. 1) identifies the process of how the final n = 21 articles were included. The numbers of studies evaluating each knee condition were: early osteoarthritis n = 3, mild/moderate osteoarthritis n = 2, severe osteoarthritis n = 1, osteoarthritis (unstated severity) n = 9, chronic knee pain n = 5, and anterior cruciate ligament reconstruction n = 1 (Table 1).

### 3.2 Study quality

The quality of the articles according to the Down and Blacks tool is displayed in Table 2. Seventeen manuscripts were classified as good and four as fair. The ‘Risk of Bias’ tool therefore provided a detailed assessment of bias (Table 3). In general there was a low or unclear risk of bias for random sequence generation, blinding of outcome assessment, incomplete outcome data, selective reporting, and other bias. As expected, there was a high risk of bias from lack of allocation concealment and blinding of participants and personnel. None of the studies scored well for blinding of participants due to the nature of the interventions. Overall, the evidence presented is from articles with a low or unclear risk of bias.

### 3.3 Description of self-care exercise based interventions

All the included studies were self-management interventions with an exercise treatment component (Table 4). A variety of different self-management modalities were used ranging from goal setting [15], [18], [19], [20], [21], [22], [23], [24], [25], telephone and individual counselling [18], [19], [22], [25], [26], information provision and education (video, lectures, printed material) [15], [16], [17], [19], [20], [21], [22], [23], [24], [25], [27], [28], [29], [30], [31], [32], [33], [34], [35], [36], problem solving [20], [32], development of coping and self-efficacy skills [15], [20], [21], [24], [29], [30], to peer support and use of review sessions [17], [21], [22], [23], [25], [28], [29], [32], [33], [34], [35]. None of the studies used web based methods of delivery for their self-care intervention. All the studies used at least one of these self-management modalities, but some included at least four. The number of self-care modalities used in the intervention does not appear to result in a better long term treatment outcome. All of the studies included structured information provision and education, except for Thomas et al. [35]. In this study advice based on individual requirement was given over the telephone. In the studies that have been explicit about the content of their information and education, there were similarities in the topics included: pathophysiology, pain management, nutrition, and joint protection.

The type of exercise and mode of delivery varied across the studies but included: individualised exercise [25], general exercise [24], supervised walking [18], [19], small group exercise and fitness [27], aerobic training [28] resistance training [28], [33], combined resistance and aerobic, information on exercise with no practical sessions [22], [32], and mixed exercise [15], [17], [21], [23], [29], [30], [31], [34], [36], i.e., various combinations of: range of motion, strengthening, balance, stretching, motor control, walking, and tai chi.

The combined self-care and exercise interventions were delivered by a range of professionals: nurses [17], [24], [25], [32], [36], physiotherapists [15], [16], [21], [23], [27], [30], [31], trained exercise professionals [18], [19], [26], [28], [33], rheumatologist [22], [32], health educator [34], and trained researcher [35]. The self-care interventions were delivered either on an individual basis [15], [22], [23], [25], [30], [32], [35], in group sessions [15], [17], [20], [21], [27], [28], [30], [31], or using a combination of both [18], [19], [24], [29], [33], [34].

### 3.4 Duration and frequency of treatment

The frequency of treatment ranged from three home visits [25] or four clinic consultations [22], [23] to three times weekly treatment over 12 months [18], [19]. For treatment duration, nine of the studies had interventions that lasted a minimum of six weeks and required attendance at least once a week [15], [17], [18], [19], [20], [21], [26], [28], [31], [33]. The length of these sessions ranged from 20 min [27] to 2 1/2 h [20]. The length of the self-management programmes ranged from four weeks [27] to two years [18], [19]. Specific guidance was generally given on the prescription of the self-care programme, but when an exercise programme was prescribed the number of repetitions, sets, and method of progression were not clearly stated. Only a limited number of studies provided specific reproducible guidelines on exercise prescription and these were interventions led by activity trainers and health educators [26], [28], [33], [34] (Table 1).

### 3.5 Control groups

The most common control arm was an alternative intervention that either had a scaled down exercise component combined with self-care [18], [19], [24], [25], [28], [32], or was a treatment that contained no self-care [23], [26], [27], [33], [34]. The other type of control used was a delayed start of the self-care intervention [20]. Several studies used a usual care control group, i.e., what the clinician felt appropriate or the patient would normally receive if they were not part of this research [15], [16], [17], [21], [22], [31]. One study used a no-intervention control [35].

### 3.6 Outcomes

The outcome measures used are listed in Table 1. The WOMAC [37] was most frequently used [15], [18], [20], [21], [22], [24], [26], [27], [30], [33], [34], [35]. This is a valid and reliable osteoarthritis specific patient rated outcome measure that assesses pain, stiffness, and function in patients with knee or hip osteoarthritis. The other frequently used valid and reliable condition specific tool was the AIMS 1/AIMS 2 [18], [25], [31], [38], [39], which measures physical, social, and emotional well-being. Three studies have specifically included an outcome measure to assess self-efficacy. This is an important variable to measure, because the aim of a self-care intervention is to promote self-efficacy [19], [17], [23], [36]. The scales used are the Stanford Self-Efficacy Scale, scale K-SES [40], and Arthritis Self-Efficacy Scale [41]. Various other questionnaires and physical tests have been used, but there is no consistency on their use across studies.

### 3.7 Length of follow-up

All the studies carried out a baseline measurement, but several did no follow-up beyond completion of the intervention and were therefore not able to assess maintenance of long-term treatment effects. For those that did a long-term follow-up, there was no standardised time frame used and this varied from two to 12 months post-completion of the intervention (Table 1). For a trial using chronic conditions this may need to be at least six months to understand the course of the condition after treatment and to allow for any immediate improvement that may be due to the Hawthorne or placebo effect [42], [43].

### 3.8 Findings

Nine studies measured outcome only up to the completion of the intervention, of these six were found to have a statistically significant improvement in outcome in the self-care intervention compared to the control group for WOMAC [15], [19], [22], [35], SF-36 [19], AIMS/2 [19], [31] and self-reported physical disability and performance test [28]. Three studies found no statistically significant difference in primary outcomes compared to the control on completion of the intervention [23], [26], [33] (Table 1).

Four studies that included long-term follow-up beyond the completion of intervention demonstrated statistically significant improvement in the self-care intervention group compared to the controls [20], [27], [34], [36]. In six studies with long-term follow-up there was no statistically significant difference between the self-care and control groups [16], [21], [24], [25], [30], [32]. In one study there was no significant improvement for the primary outcome but there was for exercise health beliefs [21]. Two studies demonstrated better outcome for self-care at completion of their intervention [15], [31], but this benefit was not maintained at long-term follow-up [16], [30].

The data was explored to evaluate if a sub-group analysis could be carried out using pain as an outcome measure across the studies. This evaluation indicated that there was too much heterogeneity (clinical diversity) between the studies based on long and short term clinical effectiveness for types of self-care and exercise interventions, professional delivering and type of control group. Therefore no meta-analysis has been carried out on this data.

## 4 Discussion

This systematic review evaluated the clinical effectiveness of self-care and exercise programmes for individuals with knee conditions. This has been done following the guidance set out in the PRISMA statement. Overall study quality was good, but based on the risk of bias tool there was an ‘unclear’ risk of bias that introduced some weakness in the evidence presented. As expected none of the studies scored well for blinding of participants due to the nature of the interventions.

When self-management and exercise outcome had been assessed at the post-intervention time point, the majority of studies demonstrated that individuals in the self-care and exercise group had a better outcome than controls [15], [18], [19], [22], [28], [31], [35]. This benefit was not maintained in studies that had a longer time span for follow-up (beyond the intervention), as only four demonstrated a long-term benefit to the patient for self-care and exercise programmes [20], [27], [34], [36]. This is important because it is the long-term success of an intervention that is important to patients and policy makers. Therefore, based on the findings of this review, there is conflicting evidence regarding the long-term effect of self-care and exercise interventions. It is recommended that in the future all studies include a long-term follow-up beyond completion of the intervention. Some of the differences in outcome between studies may be related to study design and this is discussed in the following sections.

One reason for inconsistent long-term outcome between studies may be related to the type of control group used. Three of the studies with a positive long-term outcome used a control group that was very different from the self-care intervention group, i.e., medication control, electrotherapy, delayed start, and usual care. In these studies it also appeared that individuals in the control group had less contact with the individual delivering the programme. For example, in the study by Yip et al. [17] patients in the control group received usual care, but this could have been little or no treatment and therefore no professional contact; unfortunately this was not defined in their publication. On the other hand, the studies that demonstrated no difference in outcome at the end of the intervention in the experimental group compared to the control group/s, often used treatment modalities within the different study arms that were common to the experimental and control arms. For example, in the study by Thomee et al. [23], both groups used the same pool of exercises but the self-management intervention had two sessions on self-management. In two other studies there was a distinct self-management and exercise group but both studies contained a further group that was a mixture of both exercise and self-care [26], [33]. This may have made it more difficult to demonstrate a statistically significant difference between the groups because the interventions they received were not sufficiently different to have a treatment effect. These potential confounding factors could have reduced the internal validity of the studies. It is essential in future studies to ensure the treatment content of the control group is sufficiently different to that of the experimental group.

A further factor that explains the varied clinical effectiveness of the self-care programmes is the high level of heterogeneity in the study population, research design, and intervention content. The high level of heterogeneity is the main reason that a meta-analysis had not been carried out. This review specifically included self-care interventions with an exercise component for all knee conditions to evaluate effectiveness of the interventions for enabling recovery and chronic condition prevention, as well as chronic condition management. Despite this, the majority of studies had been carried out using either an osteoarthritis or chronic knee pain population. In addition, within these populations there is a high degree of heterogeneity, especially for osteoarthritis, where it is recognised that there are different stages to the disease [44]. This means that an early osteoarthritis population is not automatically comparable to late stage osteoarthritis and heterogeneity within the population should be taken into consideration when designing the study. Adopting standardised criteria for the diagnosis of knee osteoarthritis such as that recommended by the American College of Rheumatologists [45], [46] has not routinely been used but may improve study quality and assessment of participant heterogeneity in the future.

It is evident that there is a wide range of treatment approaches available to support self-care and it needs to be established which components are most beneficial and what is the most effective and efficient manner of delivery. This systematic review can provide some insight into this by analysing in detail the group of studies that had a positive long-term effect beyond completion of the intervention. All of these studies had a well-defined information provision component, but the exercise component was either part of the information and education [20], [34], or delivered as a practical exercise group [17], [27]. Generally, there was insufficient detail on exercise prescription to be reproducible. One of these studies took self-management beyond information provision and also focused on other practical steps to develop self-management skills, such as goal setting and self-efficacy [20]. Recommendations based on this group of studies are that information provision is an essential component, but the best mode of delivery and content could not be specified. Likewise, exercise had been delivered in several ways across these studies, either through practical groups or as part of structured programmes that focus on the development of self-management skills. The optimal method is however yet to be established. Three of these studies did use the same theoretical framework [17], [20], [34], i.e., social and cognitive theory [47], to underpin their self-care approach, but there is no gold standard as to what self-management techniques this should include. In addition there is not an underlying framework that cohesively brings together the self-care and exercise components. What is reassuring is that these programmes were delivered with relatively few contacts with a healthcare professional (four to six contacts) and therefore had relatively low use of healthcare resources. This does demonstrate the potential for self-management and exercise programmes to be delivered independently to clinic visits.

A range of healthcare and exercise professionals were involved in delivering treatment across these studies. The studies with the best outcome were not delivered by one specific professional group, therefore which professional delivers the intervention does not appear to be a factor influencing clinical effectiveness. What seems essential is that adequate training is available to ensure that the individual has the skills to ensure delivery on both the self-management and exercise components. Of note, the studies that provided most detail on exercise prescription and progression were delivered by activity trainers and health educators. To improve programme quality, exercise prescription needs to be incorporated into future interventions and be embraced by other professional groups that frequently deliver exercise programmes within healthcare settings. Individuals with knee osteoarthritis, who acknowledge the importance of exercise in their management, have reported concerns over how this should be done long-term [48].

Taking into consideration the heterogeneity in populations and different treatment components within the interventions, there is unlikely to be one type of self-management and exercise combination that suits all. There is therefore a need to establish which treatment components are likely to deliver maximum benefit to individual presentations, and future research needs to be directed at phenotyping or stratifying patients accordingly to provide these answers [44]. In addition further exploratory research is required to understand the patient's perspective on the most effective self-care components and if current methods of delivering exercise meets their needs. This fits in the MRC framework for researching complex interventions [49].

The use of modern technology has not been reported by any of these studies, but using the Internet to deliver self-care programmes has been identified as an approach that warrants further research for healthcare delivery [50]. For example, online approaches could provide virtual contacts through tele-rehabilitation, better access to up to date information, virtual support groups, notifications and prompts, self-monitoring, and tracking. Several studies have recently been developed in this field, but did not match the inclusion criteria of this systematic review [51], [52]. In addition, technology could facilitate new self-care approaches that incorporate factors that individuals with long-term conditions identify as important. This includes: better support that acknowledges the physical and emotional hard work of self-care, facilitates ongoing care, does not isolate healthcare to one time point, supports individualised strategies, promotes self-efficacy, does not trivialise the condition, provides encouragement and endorsement from clinicians and introduces help to younger populations [48], [53], [54], [55].

The patient rated outcome measure most frequently used across the studies was the WOMAC, because this is an osteoarthritis specific tool which measures patient rated changes in symptoms and function, which are generally considered to be the outcomes for arthritis research [10]. This outcome measure is not transferable to other knee pathology. Interestingly, only two studies included a measure of self-efficacy, this is surprising as this is what self-care interventions target and therefore ability to self-care would be expected to improve when symptoms may not [56]. These scales [40], [41] have not undergone full psychometric testing and tend to be condition specific so they do not translate across all knee conditions. Inclusion of a self-efficacy or empowerment outcome measure needs to be considered in future research to allow comparison across studies. As yet no gold standard for use across patient groups can be recommended [56].

## 5 Conclusion

The studies included in this review demonstrated an ‘unclear’ risk of bias and conflicting evidence regarding the long-term effect of self-care and exercise interventions. Nine of the included studies failed to have a long-term follow-up, which threatens the external validity of their findings. The four studies that did demonstrate long-term clinical effectiveness all used an OA population and had a strong focus on information provision, goal setting, and developing self-management skills. The exercise component of these interventions was poorly developed and could be strengthened by improving the exercise content, prescription, and progression. The evidence on exercise prescription needs to have a higher priority alongside self-care interventions. Further research on how to combine and integrate the self-care and exercise components is required and using better designed studies on other knee conditions. This could be achieved using modern technology which to date has been underutilized in this field. Alongside this, there is a need to ensure that all healthcare professionals working in a rehabilitation environment have the skills to deliver on both the self-care and exercise treatment components. Greater integration of outcomes that measure patient ability to self-manage is required. Little evidence exists on the combined use of self-care and exercise interventions for prevention of chronic knee conditions, which needs to be addressed in the future.

## Abbreviations

AIMS 1/AIMS 2: Arthritis Impact Measurement Scale
K-SES: Knee Self-Efficacy Scale
WOMAC: Western Ontario and McMaster Universities Arthritis Index

## Table abbreviations

ACR: American College of Rheumatologists
Combined: combined intervention of self-management, exercise and control treatment
CONT: control
Exs-only: exercises intervention only
OA: osteoarthritis
SM-aerobic exs: self-management and aerobic exercise intervention
SM-exs: self-management and exercise intervention
SM-home exs: self-management and home exercise intervention
SM-home + tel: self-management, home exercise and telephone intervention
SM-only: self-management intervention only
SM-resistance exs: self-management and resistance exercise intervention
SM-tel: self-management and telephone intervention
VAS: visual analogue scale

## Figures and Tables

**Table 1:** Details of the patient populations, outcome measures, length of follow-up, and main findings for each of the studies included.

Author	Population and inclusion criteria	Study groups	Length of follow-up	Outcome measures	Main findings
Bezalel et al. [27]	Knee OA. Classified by ACR. No surgery/injection in 3 months, no other pathology affecting. 50 subjects.	SM-exs CONT	Post-intervention and 2 months post-intervention.	WOMAC Get Up & Go	2 months post-intervention: Statistically significant improvement SM-exs versus CONT p < 0.01; WOMAC total; − 9.0 (− 14.5 to − 3.4); WOMAC pain − 2.0 (− 3.7 to − 0.3); WOMAC disability − 5.9 (− 10.1 to − 1.7); Get Up & Go − 2.1 (− 3.7 to − 0.6).
Brosseau et al., part 1 [18]	Mild to moderate knee OA. ACR guidelines. 222 subjects.	SM-exs Exs only CONT	12 and 18 months	Adherence (log books) Stanford Self Efficacy Scale	18 months: No difference for total adherence rate between groups (p > 0.05). At 12 months no difference between any groups for the Stanford Self Efficacy Scale (p > 0.05). At 18 months CONT demonstrated the highest confidence about doing things from ASE (p = 0.040).
Brosseau et al., part 2 [19]	Mild to moderate knee OA. 222 subjects.	SM-exs Exs only CONT	12 and 18 months	AIMS 2 SF-36, WOMAC	At 18 months: SF-36: Statistically significant improvement: Exs-only versus SM-exs for physical functioning (p = 0.02) and pain index (p = 0.000); CONT versus exs-only and SM-exs for physical functioning (p = 0.01); pain index (p = 0.000) and standardised physical component (p = 0.002). AIMS 2: Statistically significant improvement: SM-exs versus CONT for: walking and bending (p = 0.044), household tasks (p = 0.02), symptoms component (p = 0.03), arthritis pain (p = 0.03), physical component (p = 0.01); Exs-only versus SM-exs for arthritis pain (p = 0.01), arthritis impact (p = 0.01), physical component (p = 0.04), and symptom component (p = 0.01). WOMAC: total score statistically significant improvement for CONT (p = 0.019).
Coleman et al. [20]	Knee OA. Clinical and radiological. 18 + years. Community setting. 146 subjects.	SM-exs CONT delayed start	8 weeks and 6 months	WOMAC	At 6 months: WOMAC function and total scores were statistically significantly better in SM-exs than CONT (p < 0.05) only.
Ettinger et al. [28]	60 + years, knee pain on most days, difficulty with at least one functional task due to knee pain. 439 subjects.	SM-aerobic exs SM-resistance exs SM-only	3, 9, and 18 months	Self-report physical disability Physical performance test	At 18 months: Statistically significant less disability SM-aerobic versus SM-only 1.72 (0.04) versus 1.90 (0.04) p < 0.001 and SM-resistance versus SM-only 1.74 (0.04) vs 1.90 (0.03) p = 0.003. Statistically significant improvement SM-aerobic versus SM-only on ambulation 2.22 (0.06) vs 2.63 (0.06) p < 0.01, transfer 1.75 (0.05) vs 1.92 (0.06) p = 0.02, basic activities of daily living 1.16 (0.03) vs 1.26 (0.03) p = 0.02, complex activities of daily living 1.62 (0.05) vs 1.76 (0.05) p = 0.04. Statistically significant improvement SM-resistance vs SM-only for ambulation 2.37 (0.07) vs 2.64 (0.06) p = 0.03, transfer 1.72 (0.05) vs 1.92 (0.06) p = 0.005.
Farr et al. [29]	Early onset OA. Grade 2 OA Kellgren–Lawrence. 35–68 years. 293 subjects.	SM-exs Combined: SM & Resistance CONT-resistance	3 and 9 months	WOMAC, accelerometer (moderate and vigorous physical activity)	At 9 months: No difference in WOMAC between groups. Moderate and vigorous physical activity maintained statistically significant higher in CONT-resistance than SM-exs (p = 0.034).
Hurley et al. [15]	Chronic knee pain over 6 months duration. 50 + years.	SM-exs CONT	6 weeks and 6 months	WOMAC	At 6 months: WOMAC statistically significantly better SM-exs than CONT (p = 0.01, ES 0.29). Mean WOMAC for SM-exs individual and SM-exs group statistical improvement to CONT (p = 0.04).
Hurley et al. [30]	Chronic knee pain over 6 months duration. 50 + years.	SM-exs CONT	18 and up to 30 months	WOMAC, WOMAC pain	At 30 months: No statistical difference in WOMAC function (p = 0.525) or WOMAC pain (p = 0.459).
Jessep et al. [21]	Chronic knee pain over 6 months duration. 50 + years. 64 subjects.	SM-exs CONT	6 weeks and 12 months	WOMAC	12 months: WOMAC function no difference between groups (ES 0.06; p > 0.5). SM-exs statistically significant improvement in exercise health beliefs (ES 0.6; p = 0.035).
Kovar et al. [31]	Knee OA, 40 + years, documented diagnosis of OA, 4 + months knee pain during weight bearing activities, radiographic evidence of OA. 102 subjects.	SM-exs CONT	Post-intervention (8 weeks)	AIMS, 6 minutes walk test	Post-intervention: Statistically significant improvement for: SM-exs vs CONT for walking distance (p < 0.001), AIMS physical activity (p < 0.001), and AIMS pain (p = 0.003).
Sullivan et al. [16]	As Kovar, 1992. 52 of the original 102 subjects.	SM-exs CONT	12 months	AIMS, self reported estimates of walk distance	12 months: No difference between groups for AIMS physical activity subscale (p = 0.89), arthritis impact sub-scale (p = 0.41), arthritis pain subscale p = 0.15, general health perceptions (p = 0.52), self-efficacy for pain management (p = 0.99), other symptoms (p = 0.63), or self-perceived walk distance (p = 0.17).
Mazzuca et al. [32]	Knee OA, 211 subjects, radiographic OA, recorded diagnosis of OA, Mini Mental Health Status, pharmacy record, accessible by telephone.	SM-exs CONT	4, 8, and 12 months	Health Assessment Questionnaire Disability and Discomfort Scales, Quality of Being scale.	12 months: No significant difference between groups in HAQ or quality of being scale, VAS pain (p = 0.054). Paper reports that they are significant in favour of SM-exs.
McKnight et al. [33]	Early knee OA. 35–64 years. Pain with Radiographic evidence of OA. 273 subjects.	SM-exs Exs only Combined	9 and 24 months	Leg press (maximum voluntary isometric strength), WOMAC.	24 months: All outcome measures showed a significant change over time regardless of treatment (p < 0.0001) but there was no difference between the treatment types.
Nunez et al. [34]	Patients with OA on waiting list for total knee replacement. 50–86 years. 48 subjects in each group	SM-exs CONT	9 months	WOMAC and health related quality of life	9 months: Statistically significant improvement in WOMAC function for SM-exs versus CONT (p = 0.035)
Ravaud et al. [22]	Knee osteoarthritis, 45–75 years, diagnosed according to ACR clinical and radiological guidelines.	SM-exs CONT	4 and 12 months	Weight loss, time spent on exercise (physical exercise in leisure subscale of the Baecke index), WOMAC	At 12 months: No difference between SM-exs and CONT for weight − 2.85 (4.76) vs − 2.07 (4.37) (p = 0.20). Statistically significant better physical activity score 0.23 (0.72) vs 0.08 (0.85) (p = 0.024) and WOMAC function − 867 (12.05) vs − 5.44 (12.97) (p = 0.02) for SM-exs versus CONT.
Thomas et al. [35]	Self-reported knee pain. 45 + years. 786 participants.	SM-home exs SM-tel SM-home + tel CONT	6, 12, 18 & 24 months	WOMAC	At 24 months: Combined exercise groups (SM-home exs & SM-home exs + tel) had statistically significant improvement in WOMAC pain and exercise (p < 0.001) and stiffness (p < 0.01) compared to non-exercise groups (SM-tel & CONT). No significant difference for telephone groups (SM-home exs + tel & SM-tel) and non-telephone groups (SM-home exs & CONT) for WOMAC pain (p < 0.05) or for interaction of telephone and exercise.
Thomee et al. [23]	Anterior cruciate ligament reconstruction. 40 subjects.	SM-exs CONT	4, 6, and 12 months	K-SES, Tegner physical activity, Knee Injury and Osteoarthritis Outcome Score, locus of control.	At 12 months: No statistical differences between SM-exs and CONT for any of the outcome measures (p > 0.05).
Victor et al. [24]	Knee OA. 45 + years. Clinical evidence of OA. 170 subjects.	SM-exs CONT	1 and 12 months	SF-36, general health questionnaire WOMAC	At 12 months: No significant difference in any outcomes between SM-exs and CONT.
Wetzels et al. [25]	Mild knee OA. Clinically diagnosed. 65 + years.	SM-exs CONT	6 months	AIMS 2, timed up and go	At 6 months: No statistically significant improvement in primary outcomes between SM-exs and CONT.
Yip et al. [17]	Knee OA. Medical history Criteria of American College of Rheumatology 1991. 182 subjects.	SM-exs CONT	16 weeks	ASE, VAS; pain and fatigue, self reported health, function and symptoms, health assessment questionnaire.	At 16 weeks: SM-exs statistically significant improvement SM-exs versus CONT for ASE for pain (ES 0.534) and other symptoms (ES 0.509; p = 0.0001), significant reduction in VAS pain (ES 0.613; p = 0.0001). No group difference in health assessment questionnaire.
Yip et al. [36]	Knee OA. Medical history Criteria of ACR. 182 subjects	SM-exs CONT	16 weeks and 1 year	ASE, VAS; pain and fatigue intensity, self reported health, function and symptoms.	At 12 months: Statistically significant improvement in SM-exs versus CONT for VAS pain (p < 0.01), ASE (p = 0.01), and self-rated health (p = 0.04).

**Table 2:** Quality assessment using Downs and Black score for reviewers 1 (R1) and 2 (R2). Total quality score ≥ 20 = good, 15–19 = fair, ≤ 14 = poor.

Author, year	Reporting R1	Reporting R2	External validity R1	External validity R2	Internal validity-bias R1	Internal validity-bias R2	Internal validity-confounding R1	Internal validity-confounding R2	Power R1	Power R2	R1 total quality score	R2 total quality score	Overall consensus on quality
Bezalel et al., 2010 [27]	8	10	1	1	3	5	2	4	1	1	15	19	Fair
Brosseau et al., 2012 part 1 [18]	10	9	1	1	7	7	5	3	0	0	23	21	Good
Brosseau et al., 2012 part 2 [19]	11	9	2	1	5	7	5	3	0	0	23	20	Good
Coleman et al., 2012 [20]	10	10	1	1	6	6	5	5	1	1	23	23	Good
Ettinger et al., 2006 [28]	12	10	2	2	3	6	5	4	1	1	23	23	Good
Farr et al., 2010 [29]	9	10	3	2	3	5	5	5	0	0	20	22	Good
Hurley et al., 2007 [15]	9	11	1	2	7	6	4	5	1	1	22	25	Good
Hurley et al., 2012 [30]	10	10	1	2	5	5	5	5	1	1	22	23	Good
Jessep et al., 2009 [21]	8	8	2	1	4	6	5	4	0	0	19	19	Fair
Kovar et al., 1992 [31]	11	11	2	3	4	5	3	4	0	0	20	23	Good
Sullivan et al., 1998 [16]	10	9	2	3	6	6	3	3	0	0	21	21	Good
Mazzuca et al., 1997 [32]	10	12	1	2	5	6	4	4	0	0	20	24	Good
McKnight et al., 2010 [33]	11	10	1	2	5	5	5	4	1	1	23	22	Good
Nunez et al., 2006 [34]	10	9	2	2	5	4	3	4	1	1	21	20	Good
Ravaud et al., 2009 [22]	10	11	1	3	6	7	6	3	0	1	23	25	Good
Thomas et al., 2002 [35]	8	10	2	2	6	6	4	3	1	1	21	22	Good
Thomee et al., 2010 [23]	7	7	2	1	3	5	3	3	1	1	16	17	Fair
Victor et al., 2005 [24]	9	10	4	3	5	7	3	4	1	1	22	25	Good
Wetzels et al., 2008 [25]	10	9	3	2	5	6	3	4	1	1	22	22	Good
Yip et al., 2007 [17]	9	9	2	2	4	4	4	4	1	1	20	20	Good
Yip et al., 2008 [36]	8	8	2	1	3	5	4	3	1	1	18	18	Fair

**Table 3:** Summary of study quality according to the Cochrane Risk of Bias Tool, with (+) low risk of bias, (?) unclear risk of bias, (−) high risk of bias.

Author, year	Random sequence generation	Allocation concealment	Blinding of participants and personnel	Blinding of outcome assessment	Incomplete outcome data	Selective reporting	Other bias
Bezalel et al., 2010 [27]	?	−	−	+	?	?	−
Brosseau et al., 2012 part 1 [18]	+	−	−	+	?	+	+
Brosseau et al., 2012 part 2 [19]	+	−	−	+	?	+	?
Coleman et al., 2012 [20]	?	−	−	+	?	?	+
Ettinger et al., 2006 [28]	+	−	−	+	+	+	?
Farr et al., 2010 [29]	+	−	−	−	+	+	−
Hurley et al., 2007 [15]	+	?	−	+	?	+	?
Hurley et al., 2012 [30]	+	?	−	+	+	+	+
Jessep et al., 2009 [21]	?	?	−	+	+	+	?
Kovar et al., 1992 [31]	?	−	−	−	+	+	?
Sullivan et al., 1998 [16]	?	−	−	−	+	?	−
Mazzuca et al., 1997 [32]	−	−	−	?	+	+	?
McKnight et al., 2010 [33]	+	−	−	−	+	+	?
Nunez et al., 2006 [34]	?	−	−	−	+	+	?
Ravaud et al., 2009 [22]	+	+	?	−	+	+	+
Thomas et al., 2002 [35]	?	−	−	+	−	?	?
Thomee et al., 2010 [23]	?	−	−	−	?	+	−
Victor et al., 2005 [24]	?	−	−	+	−−	?	?
Wetzels et al., 2008 [25]	+	−	?	+	?	+	+
Yip et al., 2007 [17]	?	−	−	−	?	+	?
Yip et al., 2008 [36]	?	−	−	−	?	+	?

**Table 4:** Details of the interventions for each of the studies included.

Author	Self-care interventions	Control intervention (CONT)	Duration and frequency	Instructor/facilitator
Bezalel et al. [27]	SM-exs: Exercise group. Lecture × 1: content about OA and exercise Exercise hand out Exercise diary	CONT: Short wave diathermy.	SM GP: 4 weeks, once a week, 45 min Control: 6 sessions, 20 min	Physiotherapist
Brosseau et al., part 1 [18]	SM-exs: Supervised walking programme. Behaviour intervention: Goal setting, instructor educational component, monthly face to face counselling, and telephone counselling. EXS only: Supervised walking intervention and pamphlet only.	CONT: Educational pamphlet on walking and OA.	SM: 12 months, 3 × walk week, 30 min each Behavioural component: 20 × 2 hours group sessions. Months 1–6 face to face goal setting and counselling, months 6–12 telephone counselling	Trained physical activity specialist
Brosseau et al., part 2 [19]	SM-exs: Supervised walking programme. Behaviour intervention: Goal setting, instructor educational component, monthly face to face counselling, telephone counselling. EXS only: Supervised walking intervention and pamphlet only.	CONT: Educational pamphlet on walking and OA.	SM: 12 months, 3 × walk week, 30 min each Behavioural component: 20 × 2 hours group sessions. Months 1–6 face to face goal setting and counselling, months 6–12 telephone counselling	Trained physical activity specialist
Coleman et al. [20]	SM-exs: Small groups. Holistic approach addressing: OA, self-management skills (goal setting, problem solving, modelling, positive thinking and improving self-efficacy), medication, fitness and exercise, joint protection, nutrition, fall prevention, environmental risks, and coping with negative emotions (guided imagery, cognitive behavioural therapy). Printed information.	CONT: Delayed start.	SM: 6 weeks Group session once a week, 2.5 h	Healthcare professional delivered
Ettinger et al. [28]	SM-Aerobic exercise (SM-aerobic): 3 months supervised group walking programme on indoor track. 15 months home-based walk programme: Transition phase (months 3–6) 4 home visits to develop walk programme in home environment and 6 telephone calls with leader. Maintenance Phase: (months 6–9) telephone contacted by telephone every 3 weeks (months 6–9), then monthly 10–18 months. Aerobic session: combined stretching, walking phase with callisthenics walk at 50 –70% heart rate. SM-resistance exercise (SM-resistance): 3 months facility based classes, trained leader, 15 months home-based programme, same contacts as other interventions. There were 2 orientation classes (1 h), 9 exercises, 2 sets 9 repetitions, 3 days per week for 18 months. General muscular fitness designed to strengthen major muscle groups, upper and lower limbs. Resistance progressed in stepwise manner as long as participants could complete 2 sets of 10 reps. During home phase weights exchanged at request, after face-to-face contact or telephone follow-up. Participants maintained exercise log. SM-only: Group sessions 3 × 1.5 hours sessions led by nurse. Video on OA topics: physical activity, exercise, question & answer session. Social period of 15–20 min. Printed educational material. Months 4–6 telephone contact biweekly. Months 7–18 structured telephone call about concerns and health status.	No CONT group.	18 months programmes (SM-aerobic): 3 months facility based programme once a week group exs supervised. Then 15 months home based exs. (SM-resistance): 3 months facility based programme, 1 h. Home based programme started after 2 orientation sessions, then done 3 × weekly (2 sets, 12 × reps 9 exercises) for 18 months.	Trained exercise leader
Farr et al. [29]	SM-exs: Targeted coping skills. Promoting use of adaptive strategies and fewer avoidance or passive strategies. Targeted self-efficacy through educational and behavioural techniques. Self-efficacy skills focused on increasing perceptions of control for physical functioning, pain and other OA symptoms. Structured telephone intervention to reinforce SM skills. Combined-SM-exs & CONT: altered to ensure same contact time.	CONT: Stretching and balance, ROM, isotonic muscle strength, aerobics. Progress resistance from body weight and theraband to free and machine weights. Start 1 set 6 reps 50% repetition maximum. Training logs.	SM: 9 months 12 × weekly group sessions, 90 min 24 weeks telephone intervention CONT: 9 months 3 × weekly group training	Certified Physical Activity Trainers
Hurley et al. [15]	SM-exs: Escape content: self-management, coping and education sessions and individualised supervised exercise programme. Discharged with encouragement to continue. Self-management component: Sessions covering programme overview, exercise, personal objectives and goal setting, action plans, home exercise programmes, pacing, drug management, pain theories, action plan review, managing pain, advanced home exercise, relaxation and community exercise. Mode of delivery: discussion, action plans, individual reflection/consideration and practical. Exercise component: 14 exercises addressing range of motion, strength, balance and co-ordination and aerobic fitness. Progressed as appropriate.	CONT: Usual care (what physician considers appropriate).	12 sessions, 2 × weekly for 6 weeks. Self-management component 14–20 min, exercise component 40–45 min.	Physiotherapist
Hurley et al. [30]	SM-exs: see Hurley et al. [15] above	CONT: See Hurley et al. [15] above.	See Hurley et al. [15] above	Physiotherapist
Jessep et al. [21]	SM-exs: Described above but included a physiotherapist led discussion then self paced progressive exercise circuit. Participants were given a home exercise programme and a follow-up telephone call and review session to reinforce key messages and check exercises.	CONT: Normal physiotherapy care (up to 10 sessions of what physiotherapist thought was best).	10 sessions, 15–20 minutes physiotherapist led discussion then 40 minutes self-paced progressive exercise circuit. 4 months telephone follow-up. 1 × review session	Physiotherapist
Kovar et al. [31]	SM-exs: Walking and education sessions. Group exercise (stretching and strengthening), lectures (medical aspects of osteoarthritis and exercise), discussion on barriers and benefits of walking, instruction on proper walking technique, supportive encouragement. Instructional guidebook. All based on patient needs assessment and literature review.	CONT: Routine care, called on a weekly basis to discuss activities of daily living.	SM: 8 weeks 3 × weekly, 90 minutes group (up to 30 minutes walk)	Physiotherapist
Sullivan et al. [16]	Telephone follow-up at 12 months of participants in Kovar et al. [31] study.	See Kovar et al. [31].	See Kovar et al. [31]	Physiotherapist
Mazzuca et al. [32]	SM-exs: Individualised arthritis self-care instruction based on needs. Core content: exercise (quadriceps strengthening), control of joint pain, joint protection. Identify vocational or activity most threatened by knee OA, followed by problem solving exercise and plan for maintaining this activity in ways to minimise stress on knee but maintain patient benefit from performing. Given handbook OA and exercises. Telephone contact to: 1. Assess compliance with self-care recommendations and reinforcement, 2. Clarify misconceptions, 3. Encourage continued participation.	CONT: Video about OA, OA newsletter, telephone follow-up as SM-exs.	Contact time range 30–60 min. Telephone week 1 and 1 month, calls 5–10 min.	Arthritis Nurse educator Rheumatologist supervising
McKnight et al. [33]	Exs only: Two phases: Phase 1: Supervised stretching, balance, range of motion, flexibility, isotonic muscle strength (60 min). Isotonic loads increased through 3 stages (body weight, free weights, machine weights, based on initial 3 or 6 RPM). Initially 2 × 6 repetitions, progressed to 2 × 10 repetitions then weight increased. Phase 2 (15 months): Development of self-directed long term training habits: Participants contacted every 2 weeks for first 6 weeks, then every other month. Trainers recorded exercise compliance and adjusted exercise according to participant needs on a schedule to promote self-directed long term exercising habits. Participants encouraged to meet quarterly for booster sessions. SM-exs: Development of coping and self-efficacy skills. Phase 1: 12 weeks of 90 minutes classroom sessions (lectures and discussion), followed by weekly telephone calls to boost knowledge and behaviours and provide one to one problem solving discussions. Phase 2: Telephone call bi-weekly, monthly, then bimonthly. Contact reduced following a schedule. One lecture on exercise. Participant active involvement encouraged. COMBINED: SM-exs and exs only with slight adjustment to ensure equivalent time across treatment groups.		Phase 1: 9 months Phase 2: 15 months	Physical trainers Programme manager, local healthcare professionals
Nunez et al. [34]	SM-exs: Therapeutic education and functional re-adaptation and standard pain relief. Individual and group sessions accompanied with friend or relative where possible. Content: symptom, management, pathology, joint protection, recommended treatments, tables of physical exercise no burden to lower limb, knee specific exercise (strength, range of motion and motor function), and whole body exercise (strengthen and mobilise joints). Exercises taught in group session, practice at home, supervised in second group session Pain relief; paracetamol and NSAID. Exercise prescription: Increase reps to maximum of 30 repetitions, 2 × per day for knee exercises and 10 repetitions 1 × per day for other exercises.	CONT: Pain relief of paracetamol and non-steroidal anti-inflammatory drugs.	SM-exs: 3 months. 2 × individual visits 30 min (week 1 and 3 months). 2 group sessions, 90 minutes for weeks 3 and 4. 10–12 in group. Control: 2 × individual sessions with physician.	Trained Health Educator
Ravaud et al. [22]	SM-exs: Goal orientated, standardised consultation the first information about the disease and treatment, the second about exercise, and the third about weight loss. Also included tailored counselling to help achieve behaviour modification.	CONT: Usual care over 3 visits.	SM: 3 sessions	Rheumatologists
Thomas et al. [35]	Intervention groups: SM-home exercise, SM-telephone only, SM-home exercise and telephone Exercise content: Self-paced daily home based exercise programme for strength, ROM, and locomotor function daily. Graded elastic bands used to increase resistance. Mthly telephone component: Monitor symptoms and offer simple advice. Participants discouraged from talking about exercise progression.	CONT: No intervention (no contact between sessions).	SM-home exercise and telephone; SM-home exercise 2 years intervention Daily exercise 20–30 min Initial training phase was 4 visits, 30 min in first 2 months, follow-up 6 × monthly. SM-telephone only, SM-home exercise and telephone: monthly telephone contact	Trained researcher
Thomee et al. [23]	SM-exs: standardised rehabilitation training protocol and specific training in 2 × 1 h sessions on the self-efficacy concept and the clinical rehabilitation model, followed by discussion. Rehab protocol: 4 time based phases, each with a goal. Exercises graded. Exercise types: range of motion, strengthening, functional, balance and co-ordination, aerobic and sports specific exercises. Clinical rehabilitation model: four stages on how self-efficacy can be implemented during rehabilitation. Involves goal setting and regular review and amendment; demonstrate, information, feedback, encourage, challenge practice.	CONT: Standardised rehabilitation protocol only.	SM-exs: 2 × 1 h sessions	Physiotherapist
Victor et al. [24]	SM-exs: Intervention was developed using existing evidence and recommendations. The specific aims were to inform patients about OA, to increase self-efficacy through developing strategies and skills in coping with pain and exercises; improve self-esteem and quality of life through sharing experiences and group support. Individual goal setting. Structured education programme; content was clinical information about disease, medication, other treatment, exercise pacing, and pain management. Supported by a booklet. 1 individual home session to set goals and introduce personal health diary to record medication use, symptoms and progress towards goals.	CONT: Waiting list control (booklet only).	SM-exs: 4 × 1 h groups sessions 1 individual home session	Nurse
Wetzels et al. [25]	SM-exs: Self-management and education to change life style behaviour (improving mobility and physical functioning). Three parts: 1. Prepare for home visit using an OA educational leaflet to fill out level of exercise, pain, and impairment. 2. Home visit completed forms discussed. Patients gained insight in their own OA symptoms. Subsequent agreement to try to change one of four lifestyle items (weight loss, physical exercise, walking aid use, and over the counter pain medication). 3. Follow-up telephone call after 3 months to evaluate change and what was necessary to maintain the change.	CONT: Patients in control only received the info booklet.	SM-exs: 30 minutes home visit	Nurse
Yip et al. [17]	SM-exs: Standard Arthritis self-management programme [57] and added exercise. Each participant set an exercise action plan that was reinforced weekly. Types of exercise: walking, stretching, and Tai Chi. Pedometer to motivate. ASAP topics: pain/stress management, exercise, pathophysiology, medication, treatment, joint protection, nutrition, communication, and problem solving technique. Lectures kept to a minimum, used experiential and interactive methods.	CONT: Routine care.	6 weeks 1 × weekly 2 hours group session	Nurse
Yip et al. [36]	SM-exs: Standard Arthritis self-management programme [57] and added exercise. Each participant set an exercise action plan that was reinforced weekly. Types of exercise: walking, stretching, and Tai Chi. Pedometer to motivate.	CONT: Routine care.	6 weeks 1 × weekly 2 h group session	Nurse
