# Supplementary material for: The clinical effectiveness of self-care interventions with an exercise component to manage knee conditions: A systematic review
Source: Knee. 2015 Oct;22(5):360–71. doi: 10.1016/j.knee.2015.05.003 (PMC4642743; doi:10.1016/j.knee.2015.05.003)
Supplement: Supplementary file 1 — Supplementary data. [file mmc1.docx]

**Appendix 1. Search strategy example**

1. exp Self care/

2. exp Self efficacy/

3. exp Self help groups/

4. exp Internet/

5. exp Social support/

6. exp Patient education/

7. exp Telemedicine/

8. exp Behavior therapy/

9. exp Goals/

10. (self manage* or self care or self efficacy or self help).tw.

11. (self-monitoring or social networking or behavior change or behavior therapy).tw.

12. (self management education or patient education or patient information or patient advice).tw.

13. motivational interview*.tw.

14. (social support or goal setting).tw.

15. exp Knee/ or exp Arthroplasty/ or exp Osteoarthritis/

16. exp Knee injuries/ or exp Knee joint/

17. exp Anterior cruciate ligament/

18. patello femoral.tw.

19. knee dislocation.tw.

20. (medial collateral ligament or anterior cruciate ligament or osteoarthritis or arthritis or knee arthroplasty).tw.

21. (knee injury or knee surgery or knee joint or knee replacement).tw.

22. 15 or 16 or 17 or 18 or 19 or 20 or 21

23. 1 or 2 or 3 or 4 or 5 or 6 or 7 or 8 or 9 or 10 or 11 or 12 or 13 or 14

24. 22 and 23

25. randomized controlled trial.pt.

26. controlled clinical trial.pt.

27. randomized.ab.

28. placebo.ab.

29. randomly.ab.

30. trial.ab.

31. groups.ab.

32. 25 or 26 or 27 or 28 or 29 or 30 or 31

33. 24 and 32
